# Supplementary material for: End Sequence Analysis Toolkit (ESAT) expands the extractable information from single-cell RNA-seq data
Source: Genome Res. 2016 Oct;26(10):1397–410. doi: 10.1101/gr.207902.116 (PMC5052061; doi:10.1101/gr.207902.116)
Supplement: Supplemental Material [file supp_gr.207902.116_Supp_Materials.tar.gz › ESAT-esat_v0.1/doc/html/classbroad_1_1pda_1_1seq_1_1alignment_1_1_stored_alignment_stats.html]

ESAT: broad.pda.seq.alignment.StoredAlignmentStats Class Reference


ESAT


- Main Page
- Related Pages
- Packages
- Classes
- Files

- Class List
- Class Index
- Class Hierarchy
- Class Members

All Classes Namespaces Files Functions Variables Enumerator Pages

- broad
- pda
- seq
- alignment
- StoredAlignmentStats

Public Member Functions |
Static Public Member Functions |
Static Public Attributes |
Protected Member Functions |
List of all members

broad.pda.seq.alignment.StoredAlignmentStats Class Reference

|  |  |
| --- | --- |
| Public Member Functions | |
|  | StoredAlignmentStats () throws IOException |
|  | |
| void | load (File alignmentStoredStatFile) throws NumberFormatException, IOException |
|  | |
| long | getLastModifiedStoredTimeStamp () |
|  | |
| long | getLastModifiedAlignmentTimeStampAtCreation () |
|  | |
| long | getLastModifiedAlignmentIdxTimeStampAtCreation () |
|  | |
| long | getAlignmentSizeAtCreation () |
|  | |
| long | getAlignmentIdxSizeAtCreation () |
|  | |
| boolean | isAlignmentPaired () |
|  | |
| double | getGlobalRPKMConstant () |
|  | |
| Map< String, Double > | getNumMarkers () |
|  | |
| Map< String, Double > | getNumberOfReads () |
|  | |
| Map< String, Integer > | getMaskedRegions () |
|  | |
| Map< String, Double > | getLambda () |
|  | |
| Map< String, Double > | getLocalRpkmConstant () |
|  | |

|  |  |
| --- | --- |
| Static Public Member Functions | |
| static void | store (AlignmentDataModelStats alignmentStats) throws IOException |
|  | |

|  |  |
| --- | --- |
| Static Public Attributes | |
| static final String | SAVED\_MODEL\_STATS\_EXT = ".srptr.aln.stats" |
|  | |

|  |  |
| --- | --- |
| Protected Member Functions | |
| boolean | isSafeToLoad () |
|  | |
| void | setAsUnsafeToLoad () |
|  | |
| void | setAsSafeToLoad () |
|  | |
| String | getReasonForUnsafe () |
|  | |
| void | setReasonForUnsafe (String reasonForUnsafe) |
|  | |

## Detailed Description

Class that manages the storage of an AlignmentDataModelStats to permanent storage Format of the file is simple, the first line stores the timestamp of file creation the next two columns are tab separated lines containing information about the alignment and index file when the stats were stored: Size, last modified date The next line stores the global RPKM constant The next line should be a P if the alignment is paired and anything else, usually U if it is not. Then there is a line per reference sequence containing its statistics: # markers, # reads, masked regions, lambda,

Author
:   mgarber

## Constructor & Destructor Documentation

|  |  |  |  |  |
| --- | --- | --- | --- | --- |
| broad.pda.seq.alignment.StoredAlignmentStats.StoredAlignmentStats | ( |  | ) | throws IOException |

## Member Function Documentation

|  |  |  |  |  |
| --- | --- | --- | --- | --- |
| long broad.pda.seq.alignment.StoredAlignmentStats.getAlignmentIdxSizeAtCreation | ( |  | ) |  |

|  |  |  |  |  |
| --- | --- | --- | --- | --- |
| long broad.pda.seq.alignment.StoredAlignmentStats.getAlignmentSizeAtCreation | ( |  | ) |  |

|  |  |  |  |  |
| --- | --- | --- | --- | --- |
| double broad.pda.seq.alignment.StoredAlignmentStats.getGlobalRPKMConstant | ( |  | ) |  |

|  |  |  |  |  |
| --- | --- | --- | --- | --- |
| Map<String, Double> broad.pda.seq.alignment.StoredAlignmentStats.getLambda | ( |  | ) |  |

|  |  |  |  |  |
| --- | --- | --- | --- | --- |
| long broad.pda.seq.alignment.StoredAlignmentStats.getLastModifiedAlignmentIdxTimeStampAtCreation | ( |  | ) |  |

|  |  |  |  |  |
| --- | --- | --- | --- | --- |
| long broad.pda.seq.alignment.StoredAlignmentStats.getLastModifiedAlignmentTimeStampAtCreation | ( |  | ) |  |

|  |  |  |  |  |
| --- | --- | --- | --- | --- |
| long broad.pda.seq.alignment.StoredAlignmentStats.getLastModifiedStoredTimeStamp | ( |  | ) |  |

|  |  |  |  |  |
| --- | --- | --- | --- | --- |
| Map<String, Double> broad.pda.seq.alignment.StoredAlignmentStats.getLocalRpkmConstant | ( |  | ) |  |

|  |  |  |  |  |
| --- | --- | --- | --- | --- |
| Map<String, Integer> broad.pda.seq.alignment.StoredAlignmentStats.getMaskedRegions | ( |  | ) |  |

|  |  |  |  |  |
| --- | --- | --- | --- | --- |
| Map<String, Double> broad.pda.seq.alignment.StoredAlignmentStats.getNumberOfReads | ( |  | ) |  |

|  |  |  |  |  |
| --- | --- | --- | --- | --- |
| Map<String, Double> broad.pda.seq.alignment.StoredAlignmentStats.getNumMarkers | ( |  | ) |  |

|  |  |  |  |  |
| --- | --- | --- | --- | --- |
| String broad.pda.seq.alignment.StoredAlignmentStats.getReasonForUnsafe | ( |  | ) |  |

protected

|  |  |  |  |  |
| --- | --- | --- | --- | --- |
| boolean broad.pda.seq.alignment.StoredAlignmentStats.isAlignmentPaired | ( |  | ) |  |

Here is the caller graph for this function:

|  |  |  |  |  |
| --- | --- | --- | --- | --- |
| boolean broad.pda.seq.alignment.StoredAlignmentStats.isSafeToLoad | ( |  | ) |  |

protected

|  |  |  |  |  |  |
| --- | --- | --- | --- | --- | --- |
| void broad.pda.seq.alignment.StoredAlignmentStats.load | ( | File | *alignmentStoredStatFile* | ) | throws NumberFormatException, IOException |

Here is the call graph for this function:

|  |  |  |  |  |
| --- | --- | --- | --- | --- |
| void broad.pda.seq.alignment.StoredAlignmentStats.setAsSafeToLoad | ( |  | ) |  |

protected

|  |  |  |  |  |
| --- | --- | --- | --- | --- |
| void broad.pda.seq.alignment.StoredAlignmentStats.setAsUnsafeToLoad | ( |  | ) |  |

protected

|  |  |  |  |  |  |
| --- | --- | --- | --- | --- | --- |
| void broad.pda.seq.alignment.StoredAlignmentStats.setReasonForUnsafe | ( | String | *reasonForUnsafe* | ) |  |

protected

|  |  |  |  |  |  |
| --- | --- | --- | --- | --- | --- |
| static void broad.pda.seq.alignment.StoredAlignmentStats.store | ( | AlignmentDataModelStats | *alignmentStats* | ) | throws IOException |

static

## Member Data Documentation

final String broad.pda.seq.alignment.StoredAlignmentStats.SAVED\_MODEL\_STATS\_EXT = ".srptr.aln.stats"

static

---

The documentation for this class was generated from the following file:

- java/broad/pda/seq/alignment/StoredAlignmentStats.java


---

Generated on Fri Aug 15 2014 15:11:01 for ESAT by  

 1.8.7
